# Dry Matter Accumulation in Maize in Response to Film Mulching and Plant Density in Northeast China

**DOI:** 10.3390/plants11111411

**Published:** 2022-05-26

**Authors:** Zhenchuang Zhu, Shmulik P. Friedman, Zhijun Chen, Junlin Zheng, Shijun Sun

**Affiliations:** 1 College of Water Conservancy, Shenyang Agricultural University, Shenyang 110866, China; 2020200012@stu.syau.edu.cn (Z.Z.); 2018200014@stu.syau.edu.cn (Z.C.); junlinzheng@syau.edu.cn (J.Z.); 2 Department of Environmental Physics and Irrigation, Institute of Soil, Water and Environmental Sciences, Agricultural Research Organization, The Volcani Center, Rishon LeZion 7505101, Israel; vwsfried@volcani.agri.gov.il

**Keywords:** effective accumulated temperature, logistic equation, maize developmental progress, plastic film color, temperature compensation

## Abstract

Film mulching in combination with high plant density (PD) is a common agronomic technique in rainfed maize (*Zea mays* L.) production. However, the effects of combining colored plastic film mulching and PD on dry matter accumulation (DMA) dynamics and yield of spring maize have not been thoroughly elucidated to date. Thus, a 2-year field experiment was conducted with three mulching treatments (no mulching (M0), transparent plastic film mulching (M1), and black plastic film mulching (M2)) and five plant densities (60,000 (D1), 67,500 (D2), 75,000 (D3), 82,500 (D4), and 90,000 plants ha^−1^ (D5)). A logistic equation was used to simulate the DMA process of spring maize by taking the effective accumulated air temperature compensated by effective accumulated soil temperature as the independent variable. The results showed that compared with M0 treatment, the growth period of M1 and M2 treatments was preceded by 10 and 4 days in 2016, and 10 and 7 days in 2017, respectively. The corrected logistic equation performed well in the characterization of maize DMA process with its characteristic parameter (final DMA, *a*; maximum growth rate of DMA, *GR_max_*; effective accumulated temperature under maximum growth rate of DMA, *x_inf_*; effective accumulated temperature when maize stops growing, *x_max_*; effective accumulated temperature when maize enters the fast-growing period, *x*_1_). Plastic film color mainly affected DMA by influencing *x_inf_*. PD mainly affected DMA by affecting *GR_max_* and *x*_1_. During the first slow growing period, the DMA of M1 treatment was the largest among the three mulching treatments, however, during the fast growing period, the DMA of M2 treatment accelerated and exceeded that of M1 treatment, resulting in the largest final DMA(*a*) and yield. When the PD was increased from D1 to D4, the maximum growth rate (*GR_max_*) continued to increase, and the effective accumulated temperature when maize enters the fast growing period (*x*_1_) continued to decrease, which substantially increased the final DMA(*a*) and yield. The application of M2D4 treatment can harmonize the relevant factors to improve the DMA and yield of spring maize in rainfed regions of Northeast China.

## 1. Introduction

Maize (*Zea mays* L.) is the most prevalent cereal crop in China, and its yield increasing has an important role in grain production across the whole country [1]. As a typical cool, high-latitude, and rainfed area, Northeast China is one of the most important cultivated regions for cereal crops in China [2], with its spring maize planting area and yield accounting for approximately 30.9% and 34.0% of the country’s output [3]. In recent years, scarce precipitation and low temperature frequently occurred during the spring in Northeast China, representing the main limiting factors for increasing maize yield [4,5]. Appropriately increasing plant density (PD) is conducive to increasing stand leaf area index, enhancing the utilization of solar radiation, and improving maize yield and water use efficiency [6]. However, too high PD limits substantially the volume of the single plant canopy and initiates size-asymmetric competition on light, resulting in the decline of maize yield [7]. Plastic film mulching with an adequate increase in plant density can effectively increase soil surface temperature, reduce soil surface evaporation, and increase soil water content, thus alleviating the competition between neighboring plants for essential resources upon increasing PD, making the formers important agronomic measures to increase maize yield [7,8,9].

The formation of crop yield is closely related to the process of dry matter accumulation (DMA). Thus, the analysis of dynamic changes in DMA during crop growth is important for yield prediction. Different cultivation measures have different effects on DMA [10,11]. Plastic film mulching can increase the DMA [12]. However, the effect of plastic film mulching on soil water content and temperature varies with film colors [13], resulting in differences in DMA. Different PDs can also create different canopy structures and root systems that affect radiation [6] and water [14] use efficiency, thereby determining maize DMA and yield. Therefore, it is necessary to establish a simple, applicable, and universal model to accurately reflect the dynamics of DMA of spring maize in rainfed areas, especially under the conditions of plastic film mulching with different PDs.

Mathematical models such as the Richards, Logistic, and Gompertz equations are often used to simulate plant growth dynamics in terms of, e.g., plant height, leaf area index, and DMA [15,16,17,18]. Causton and Venus [19] reported that the Richards model was more effective in simulating the growth of crops such as maize and wheat, while the Logistic model is a special case of the Richards model. The parsimonious logistic model is widely used because of its few parameters and its applicability. Sepaskhah et al. [20] used the logistic model to quantitatively analyze the effect of seasonal water and fertilizer application on maize DMA and yield. Mahboh et al. [21] used the logistic model to study winter wheat DMA under different irrigation regimes and nitrogen application rates. Zhang et al. [22] used the logistic model to study the effect of increasing PD on maize DMA and dry matter distribution in maize/peanut intercropping conditions. However, little information is available concerning the application of the logistic model for the simulation of the DMA dynamics of maize under different plastic film mulching and PD treatments.

In this study, we used the logistic equation with effective accumulated air temperature compensated by effective accumulated soil surface temperature as an independent variable, and a universal yield–plant density (Y-PD) model to analyze the characteristics of maize DMA under different colored plastic film mulching and PD. The objectives of the reported study are (1) to determine the effects of different mulching treatments on effective accumulated temperature and maize developmental progress; (2) to investigate the dynamic process of the DMA of spring maize in response to different colored plastic film mulching and PD; (3) to explore the appropriate mulching and PD treatments in maize production in Northeast China.

## 2. Results

### 2.1. Effects of Different Mulching Treatments on Effective Accumulated Temperature and Maize Developmental Progress 

Plastic film mulching increased surface soil temperature (Table 1 and Table 2). The two-year data showed that the order of the accumulated effective soil temperature with different treatments were as following: M2 > M1 > M0. However, the accumulated effective air temperature with different treatments followed the following order: M0 > M2 > M1. The reason for the difference is that plastic film mulching clearly accelerated the maize growth and development progress. The growth period under M1 and M2 treatments was preceded by 10 and 4 days in 2016, and 10 and 7 days in 2017, respectively, compared with M0 treatment.

The warming effect of plastic film mulching is mainly manifested in the early growth stages (Table 1 and Table 2). From the sowing to the three-leaf stage, the effective accumulated soil temperature under the M1 and M2 treatments was 28.4 °C d (daily average 1.8 °C) and 18.1 °C d (daily average 1.0 °C) higher than that under no mulching when the time is the same as plastic film mulching (NMTSM) in 2016, respectively. The extra effective accumulated soil temperature of plastic film mulching treatments compensated for the insufficient effective accumulated air temperature. Therefore, the period of the maize sowing—three-leaf stage of the M1 and M2 treatments were shorter by 5 and 3 days, respectively, compared to that of the M0 treatment. Similarly, the effective accumulated soil temperature under the M1 and M2 treatments increased by 20.6 °C d (daily average 1.5 °C) and 18.6 °C d (daily average 1.2 °C) in 2017, respectively, compared to NMTSM, the M0, M1 and M2 treatments completed the growth from sowing to three-leaf stage in 20, 14 and 16 days, respectively. With the advancement of the growth process, the warming effect of film mulching weakened. During maize three-leaf—joining stage, jointing—tasseling stage, the effective accumulated soil temperature under the M1 and M2 treatments increased by 30.3 °C d (daily average 1.2 °C) and 18.4 °C d (daily average 0.8 °C), 18.3 °C d (average daily 0.7 °C) and 11.9 °C d (average daily 0.5 °C) in 2016, respectively, compared to NMTSM. In 2017, the effective accumulated soil temperature under the M1 and M2 treatments increased by 34.6 °C d (daily average 1.3 °C) and 23.8 °C d (daily average 1.1 °C), 26.7 °C d (daily average 1.0 °C) and 14.7 °C d (daily average 0.7 °C), respectively, compared to NMSTM. These results indicate that the effect of the M1 treatment on increasing soil temperature is stronger than that of the M2 treatment.

### 2.2. Effects of Different Treatments on Spring Maize Yield

ANOVA revealed significant effects of the different treatments on maize yield and yield components in 2016 and 2017 (Table 3). The maize yields under the M1 and M2 treatments increased by 4.63% and 7.64% in 2016, and by 4.39% and 8.28%, in 2017, respectively, compared to the M0 treatment (Table 4). The results indicated that maize yield could be increased using plastic film mulching, with the greatest improvement expected for the M2 treatment. As for the PD, the maize yields in the D4 and D5 treatments were not significantly different (*p* > 0.05); however, they were greater than that for the lower PD treatments. Specifically, the maize yield of the D4 treatment was larger than that of other PD treatments by 2.58–25.25% in 2016 and by 1.27–22.45% in 2017. In terms of yield composition, the differences of spike length, kernels per ear and 100-kernels weight under different film mulching were significant (*p* < 0.05 or *p* < 0.01) and sequenced as following: M2 > M1 > M0. All spike length, spike diameter, kernels per ear and 100-kernels weight decreased with the increase of PD.

**Figure 1:**
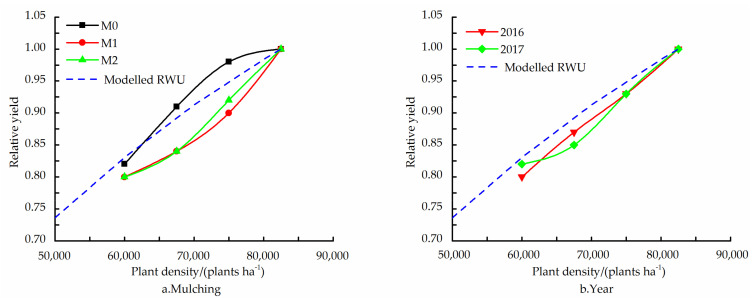
Relative yield, yield (*Y*) normalized by the maximum yield obtained at a PD of 82,500 (D4) plants ha^−1^ (*Y_max_*), as function of PD for no (M0), transparent (M1) and black (M2) film mulching treatment ((**a**), mean of 2016 and 2017), and for the two experimental years ((**b**), mean of three mulching treatments). Also shown is the *Y/Y_max_* relationship according to the fitted relative water uptake relationship.

The ability of the fitted RWU relationship to predict the general trend of dependence of the obtained yields on the PD is shown in Figure 1. For all three mulching treatments in both years, the maximum yield was obtained for a PD of the D4 treatment with a subsequent decrease at the D5 treatment (Table 4). 

The yield response to the PD was noticeably stronger for the two film mulching treatments as compared to the M0 treatment, i.e., increasing the PD in the relevant range from D2 to D4 treatment caused a greater increment in yield (both absolutely and relatively) (Figure 1a). The response of the relative yields to the PD, reliably described by *Y*/*Y*_max_ with r_0_α^−1^ = 0.0112 m^2^, was very similar for both years (Figure 1b).

### 2.3. Fitting the Logistic Equation to the DMA Curves

The logistic equation was used to fit the dry matter accumulation of spring maize in 2016 and 2017. The coefficient of determination (*R*^2^) varied from 0.89 to 0.99, and the *p*-values of all treatments were <0.05 (Table 5), i.e., the logistic equation adequately describes the DMA patterns of all treatments. 

The logistic curve parameter *k* had the largest variation range, with *C_V_* values of 14.5% and 19.1% in 2016 and 2017, respectively, i.e., different treatments have the greatest impact on the steepness of the logistic curve. Followed by the parameter *a*, with *C_V_* values of 11.6% and 16.0%, respectively; the variability of the *x_c_* values was the smallest, with *C_V_* values of 7.4% and 6.4%, respectively. These results indicated that the logistic equation, with the effective accumulated air temperature compensated by the effective accumulated soil surface temperature as an independent variable, can adequately simulate the DMA process of maize under conditions of different colored plastic film mulching and PD.

### 2.4. Effect of Different Treatments on the Dynamic Process of DMA

The plastic film color, PD and their interaction, all had significant effects on the final DMA (*a*) (*p* < 0.01 or *p* < 0.05, Table 6). In addition, plastic film color had a significant effect on the effective accumulated temperature at the maximum growth rate of DMA, *x_inf_* (*p* < 0.01). PD had a significant effect on the maximum growth rate, *GR_max_*, and on the effective accumulated temperature when entering the fast growing period, *x*_1_ (*p* < 0.05 or *p* < 0.01). The interaction between plastic film color and PD had no significant effect on *x_inf_*, *x_max_* and *x*_1_, but had a significant effect on *GR_max_* (*p* < 0.05). 

With respect to the main effects, the values of *a* under the three mulching treatments followed the following order: M2 > M1 > M0 (Table 7). The values of *x_inf_* under the M1 and M2 treatments were similar, and they were higher than that of the M0 treatment. The value of *a* increased with the increasing PD in 2016. However, it first increased and then decreased with the increase of PD in 2017. The maximum value of *a* was obtained in D3 treatment. *GR_max_* increased with the increasing PD, with a substantial increase seen from D1 to D3 treatments, but not from D3 to D5 treatments. The value of *x*_1_ firstly decreased and then increased with the increasing PD, while it was the lowest for the D4 treatment (54.6–145.8 °C d in 2016, 17.1–43.6 °C d in 2017, respectively, lower than that for the other PD treatments).

**Figure 2:**
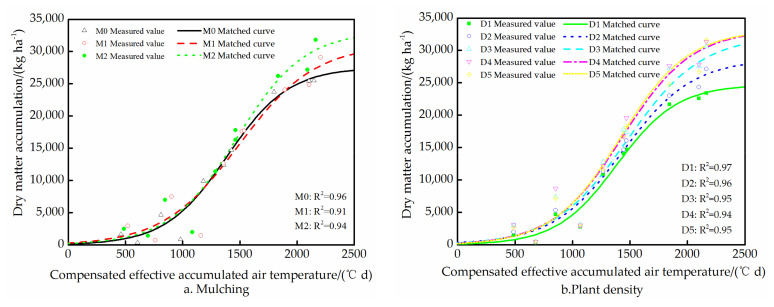
The measured value of DMA (average of each treatment in 2016 and 2017) and the fitted growth curve of DMA under film mulching (**a**) and PD (**b**).

DMA showed a trend of “slow–fast–slow” growth process. In the first slow growth period, the DMA under the three mulching treatments followed the order: M1 > M2 > M0 (Figure 2a). However, in the fast growth period, from the jointing to the filling stage, the DMA under the M2 treatment accelerated, exceeding that under the M1 treatment. The film treatments increased the time of the fast growth period; the DMA under the M0 treatment entered the final slow growth period earlier than the M1 and M2 treatments. The PD affected the whole growth process (Figure 2b). From the D1 to the D4 treatments, DMA increased with PD, no matter in the two slow growth periods or in the fast growth periods, with the DMA curves for the D4 and D5 treatments practically coinciding, indicating that increasing PD beyond that of the D4 treatment did not significantly increase the DMA.

### 2.5. Effect of Different Treatments on the Dynamic Characteristics of DMA

The *a* and *GR_max_* were significantly affected by the PD and mulching (Table 6), thereby representing the two most relevant parameters reflecting the dependence of soil water availability on the PD. Therefore, the a priori unknown interplant competition factor (4π*r_0_α*^−1^) of the universal *Y/Y_max_* relationship was evaluated by simultaneously fitting (via a minimum-least-squares procedure) the following two analogous expressions for *a*(*ρ*) and *GR_max_*(*ρ*):

*a*/*a* (*ρ*→∞) = 1 − exp(4*πr*_0_*α*^−1^*ρ*) (1)

*GR_max_*/*GR_max_* (*ρ*→∞) =1 − exp(4*πr*_0_*α*^−1^*ρ*) (2)

**Figure 3:**
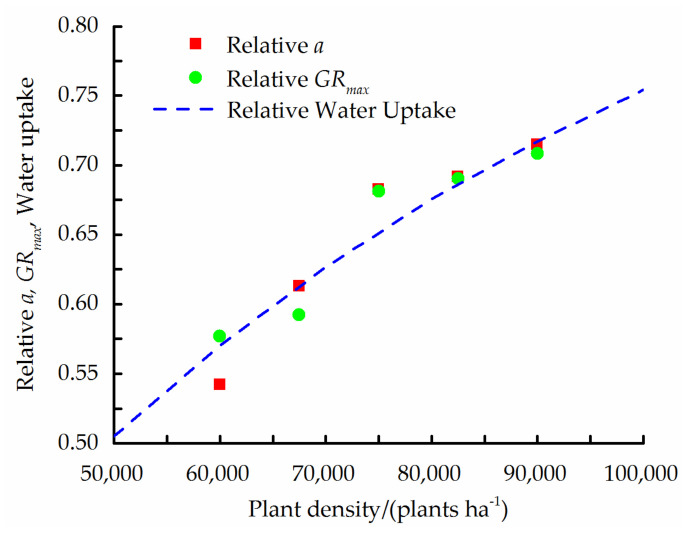
Relative water uptake as function of PD (broken line) fitted to the dependence of the final DMA, *a* (square markers), and of the maximum growth rate, *GR_max_* (circular markers), growth model parameters on the PD.

The fitted values were *r*_0_*α*^−1^ = 0.0112 m^2^, *a*(*ρ*→∞) = 46,813 plants ha^−1^, and *GR_max_*(*ρ*→∞) = 49.3 kg (ha °C d)^−1^, and the fitted relative water uptake expression (1−exp(4π*r*_0_*α*^−^^1^*ρ*)), along with the relative a and *GR_max_* parameters, are depicted in Figure 3.

As an example, if the expected soil capillary length (*α*^−1^) is about 0.1 m for the top 40 cm of the silty loam soil in the experimental field (), according to the fitted value of 0.0112 m^2^ for *r*_0_*α^−^*^1^, the mean radius of the maize root system (disregarding its dependence on PD) would be about 0.112 m, i.e., most of the water uptake would take place in the top 22.4 cm of soil.

**Figure 4:**
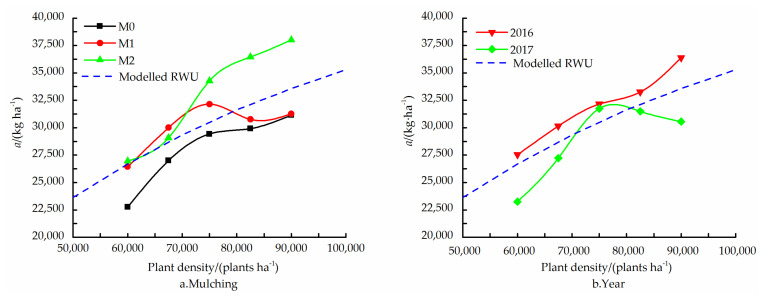
Final DMA (*a*) as function of PD for no (M0), transparent (M1) and black (M2) film mulching treatment ((**a**), mean of 2016 and 2017), and for the two experimental years ((**b**), mean of three mulching treatments). Also shown is the *a*(*ρ*) relationship according to the fitted relative water uptake relationship.

The multivariate analysis of variance revealed a significant interaction (*p* < 0.05) between the effects of mulching treatments and PD on *a*. This can also be seen in Figure 4a, which shows that, for the M0 and M1 treatments, an increase in the PD beyond D3 treatment caused a modest, if at all, increase in *a*, while, for the M2 treatment, there was a substantial increase in *a*. There was considerably more rain in the growing season of 2016 compared to the 2017 season (). This is probably the reason why *a* reached a maximum value at D3 treatment in 2017, but continued to increase with an increase in PD in 2016 (Figure 4b).

**Figure 5:**
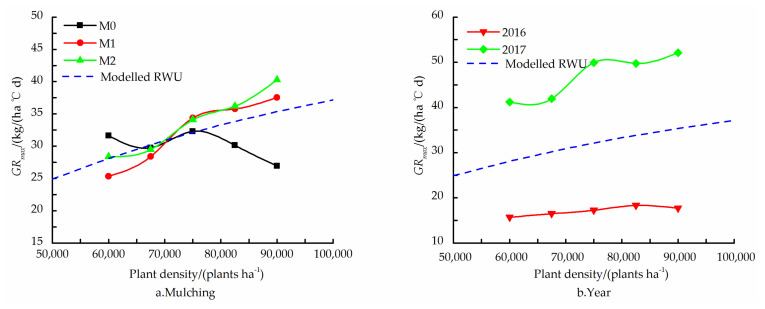
Maximum growth rate (*GR_max_*) as function of PD for no (M0), transparent (M1) and black film (M2) mulching treatment ((**a**), mean of 2016 and 2017), and for the two experimental years ((**b**), mean of three mulching treatments). Also shown is the *GR_max_*(*ρ*) relationship according to the fitted relative water uptake relationship.

A similar analysis of the interaction between the effects of mulching treatments and PD on *GR_max_* revealed that, while, for the two film mulching treatments, *GR_max_* increased steeply with an in increase in PD, for the M0 treatment, the effect of PD was opposite (at least in the range of D3 to D5 treatment), i.e., the *GR_max_* decreased with increasing PD (Figure 5a). The *GR_max_* was much higher in 2017 compared to 2016, albeit increasing with PD in both years (Figure 5b).

### 2.6. The Relationship between the Final DMA(a) and the Dynamic Characteristics of DMA

In order to further analyze the influence of the characteristic parameters of the dynamic process of DMA on *a* of spring maize, a path analysis was performed (Table 8). The direct path coefficients show that the order of influence on *a* is *GR_max_* > *x_inf_* > *x_max_* > *x*_1_. *GR_max_* had the greatest effect on *a* (*p* < 0.01), whereby the contribution to *R*^2^ was 0.9669, indicating that a greater maximum growth rate results in a greater DMA. The second most influential factor was *x_inf_*, indicating that a larger effective accumulated temperature when reaching the maximum growth rate results in a greater DMA. Finally, the correlation coefficients of *x_max_* and *x*_1_ were negative, indicating that a smaller effective accumulated temperature when growth stops or when entering the fast growing period results in a greater DMA; however, the influence was weak.

## 3. Discussion

### 3.1. Accumulated Temperature under Plastic Film and Model Application 

Earlier studies revealed that plastic film mulching can increase soil temperature, thereby improving crop growth [12]. In our study, the transparent plastic film mulching (M1) was more effective in increasing soil temperature than the black plastic film (M2), which is due to that, on the one hand, the transparent film can transmit more solar radiation and reduce the reflection of solar radiation [23], and on the other hand, the long wave radiation can be blocked by the dew condensation on the transparent film, causing the soil temperature to rise. The higher soil temperature promotes the growth and development of maize. Previous studies have suggested that plastic film mulching clearly reduced the number of days required for maize to ripe [24]. In our study, the increasing soil temperature by plastic film mulching is mainly manifested in the early growth stage, which eliminates the damage of low temperature and chill damage in the spring. This is the reason for reducing the number of days to germination and enhancing early seedling growth, with the greatest improvement seen for the M1 treatment.

In early applications, the logistic model, which typically uses time as the independent variable, was used to describe the plant growth process [25]. Sepaskhah et al. [20] suggested replacing the time variable with ecological factors such as accumulated temperature (degree-days), as it better reflects maize growth. Behind the use of accumulated temperature is the notion that the growth rate of crops is mainly affected by temperature; in other words, only when the accumulated temperature reaches a certain value can a certain growth stage be accomplished [26]. Under the same accumulated air temperature, the maize growth rate with plastic film mulching is obviously faster than under that with no mulching, somewhat contradicting the accumulated temperature concept. The compensation effect of accumulated soil temperature on accumulated air temperature [27] with plastic film mulching explains this apparent contradiction. In the present study, a logistic model using effective accumulated air temperature compensated by effective accumulated soil temperature as the independent variable was adopted. The results (*p* and *R*^2^ values) show that the logistic curve could adequately simulate the process of DMA under different colored plastic film mulching and PD conditions. 

### 3.2. Dry Matter Accumulation Dynamics 

The plants were relatively short, and there was no significant difference in ground shading among density treatments at the seedling stage of spring maize. While after seedling stage, high-density treatment formed a larger canopy structure, which led to the increase in ground shading and the decrease in soil temperature [13]. However, the leaf area index increased with an increase in PD, such that more photosynthetically active radiation could be intercepted, thereby leading to an increase in DMA [28]. Moriri et al. [29] also stated that with an increase in maize PD, the DMA increased. We found that *GR_max_* increased significantly with the increase in PD from the 60,000 (D1) to the 75,000 (D3) plants ha^−1^ treatments, but it did not increase significantly with a further increase in PD. The value of *x_l_* firstly decreased and then increased with the increase in PD, being lowest for the 82,500 plants ha^−1^ (D4) treatment (Table 7). Consequently, when the PD increased from the D1 to D4 treatments, the DMA increased gradually, whereas a further increase in PD did not significantly increase the DMA (Figure 2b). This is so because the photosynthetic rates of the middle-height and lower leaves of the plant are reduced, resulting in fewer photosynthetic products per leaf (area) under high-density conditions [6], with a lower DMA per plant. If the increase in PD makes up for the decrease in dry matter production per plant, the DMA per area would increase; otherwise, the areal DMA would reach a plateau or even decrease. Dang et al. [30] found that plastic film mulching can conserve soil water, increase soil temperature in the early stages of the crop, thus accelerating crop growth and development. In our study, the effect of the M1 treatment on improving soil temperature was better than that of the M2 treatment, and the DMA under the M1 treatment was the largest among the three mulching treatments in the first slow growing period. However, the DMA under the M2 treatment exceeded that of the M1 treatment in the fast growing period, resulting in the largest *a* and yield (Figure 2a). This is because the higher soil temperature in the M1 treatment is beneficial for DMA in the early growth stages; however, it also shortens the growth period, affecting the grain-filling process. Compared with the M1 treatment, the M2 treatment could provide a better soil temperature, suitable for maize growth in all growth stages, with a good effect on soil water storage [13], Therefore, with black-plastic-film mulching, upon increasing PD beyond the D3 treatment, a still rose sharply (Figure 4). 

Usually, the correlation of water uptake with maize DMA is stronger than its correlation with grain yield [14]. The general, mean trends of *a*(*ρ*) and *GR_max_*(*ρ*) for the different mulching treatments in both years was described reasonably well by Equation (1) with *r*_0_*α^−^*^1^ = 0.0112 m^2^; of course, this was expected, as *a*(*ρ*) and *GR_max_*(*ρ*) were used to evaluate *r*_0_*α^−^*^1^. However, the ability of the fitted RWU–PD relationship to predict the general trend of dependence of the obtained yields on the PD (Figure 1) is more constructive, in accordance with the high correlation found between the fitted value of a and the measured yields.

Sun et al. [13] found that although plastic film mulching increased the costs of materials and residual film recovery, it also substantially increased the maize yield compared with no mulching, resulting in better economic benefits of the M2 treatment than that of the M1 treatment. With the extension of the use time and with the continuous expansion of its usage, while plastic film mulching brings more benefits to agricultural production, it also produces negative effects such as farmland residues and landscape pollution [31]. So far, there are two ways to solve the problem of plastic film pollution: one is to increase the recovery of plastic film, and the other is to develop and promote degradable films.

## 4. Conclusions

(1) The increase in soil temperature caused by plastic film mulching accelerated the growth and development of spring maize, resulting in a shorter growth period as compared to the no mulching control, with the greatest improvement obtained from transparent plastic film mulching (M1).

(2) The dry matter accumulation (DMA) under transparent film mulching was the largest among all the mulching treatments in the first slow growing period. However, during the fast growing period, the DMA under black film mulching was higher than that of the transparent film, resulting in the largest final DMA (*a*) and yield. Among all plant densities, the PD of 82,500 plants ha^−1^ had relative larger maximum rate of DMA (*GR_max_*) and the smallest effective accumulated temperature when entering the fast growing period (*x_l_*), thus increased *a* and maize yield.

(3) The plant density of 82,500 plants ha^−1^ combined with black plastic film mulching (M2D4) can increase the DMA and economic yield of maize in rainfed regions of Northeast China.

## 5. Materials and Methods

### 5.1. Site Description

**Figure 6:**
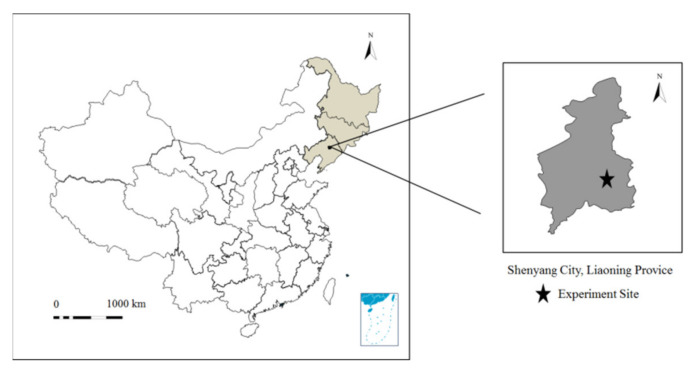
The geographic location of the study site.

**Figure 7:**
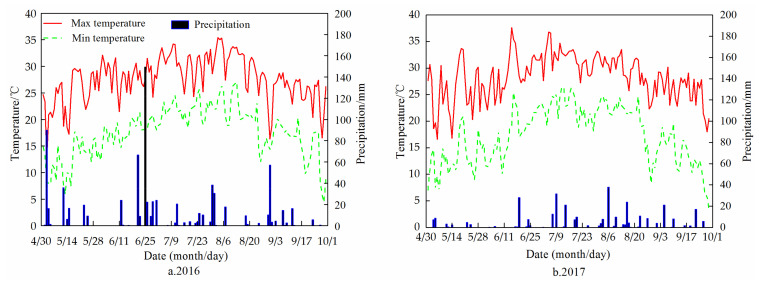
Daily temperature and precipitation during the maize growing season in 2016 and 2017.

In 2016 and 2017, field experiments were performed at the experimental station of the Water Conservancy College of Shenyang Agricultural University (41°44′N, 123°27′E), located in Shenyang City, Liaoning Province in Northeast China. The exact geographical location is shown in Figure 6. The study area is characterized by a semi-humid continental climate. The average annual precipitation is 703 mm, the average annual temperature is 8.0 °C. The monthly mean maximum and minimum temperatures of the two maize growth seasons (seeded on 1 May and harvested on 27 September in 2016; seeded on 3 May and harvested on 23 September in 2017) were 30.5 and 12.4 °C, and 31.9 and 13.2 °C, respectively (Figure 7). Precipitation during the 2016 and 2017 maize growth seasons was 790 and 305 mm, respectively. The soil of the study site is a silty clay loam (Table 9). In the 0–100 cm soil layer, the average soil bulk density is 1.41 g cm^−3^, the average (volumetric) water content at field capacity (1/3 bar) is 0.38 cm^3^ cm^−3^, and the average wilting point (15 bar) is 0.18 cm^3^ cm^−3^. Moreover, the topsoil (0–20 cm) contains 20.8 g kg^−1^ organic matter, 0.87 g kg^−1^ total N, 8.9 mg kg^−1^ available P, and 75.6 mg kg^−1^ available K. During the study period, the average groundwater depth in the experimental area was about 4.2 m.

### 5.2. Experimental Design and Field Management

**Figure 8:**
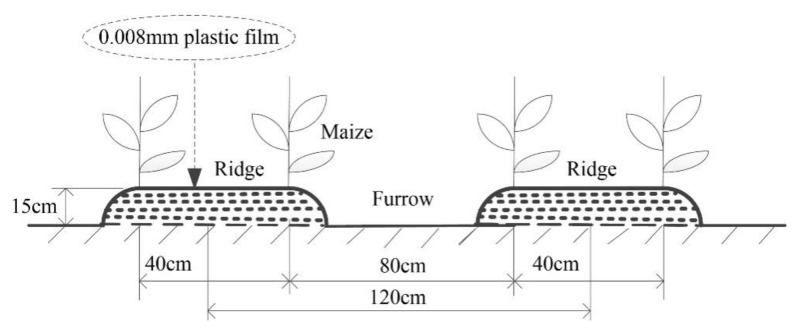
Schematic diagram of large-ridge-double-lines planting method.

A density-tolerant maize variety (cv. Liangyu 99) was used with a traditional large-ridge double-line planting method (Figure 8). The experiment was laid out as a split-plot block design. The main plot was three mulching treatments: no mulching (M0), transparent plastic film mulching (M1, 1.2 m wide × 0.008 mm thick), and black plastic film mulching (M2, 1.2 m wide × 0.008 mm thick). The split plot was five PD treatments: 60,000 (D1), 67,500 (D2), 75,000 (D3), 82,500 (D4), and 90,000 plants ha^−1^ (D5), recommended by local farmers’ practice. A total of 15 combinations were tested, and each combination was repeated three times, totaling 45 experimental plots (6.0 m × 3.6 m). Protective lines were set up around the plot, and border lines were set up between different planting modes. The plots were fertilized only once, during sowing, with a compound fertilizer that contained 243 kg N ha^−1^, 135 kg P_2_O_5_ ha^−1^, and 117 kg K_2_O ha^−1^. Field managements were in line with local farmers’ practices. No supplemental (to natural rainfall) irrigation was provided during the whole maize growing period. 

### 5.3. Measurements and Computations

#### 5.3.1. Temperature

Air temperature was obtained from a meteorological station equipped near to the experimental site.

Soil temperature at 5 cm depth was recorded using soil thermometers (Chuangji Instrument Co., Ltd., Hengshui, China). The soil thermometers were inserted vertically near the maize root system. Soil temperature was measured daily at 6:00 a.m. and 2:00 p.m.

#### 5.3.2. Maize Phenology

The exact dates corresponding to the main growth stages of maize such as the three-leaf, jointing, tasseling, silking and maturity stages were observed and recorded.

#### 5.3.3. Dry Matter Accumulation (DMA)

At the end of the seedling, jointing, heading, filling, and maturity stages, three representative plants from each plot were cut at the base of the stem, bagged according to the stem, leaf, and spike classification, inserted into an oven to de-enzyme (105 °C) for 30 min, and then dried at a temperature of 80 °C until the mass stabilized to determine the above-ground dry matter.

#### 5.3.4. Yield and Yield Components

At the end of the growing period, maize yields were measured by hand harvesting of all plants from a 10 m^2^ area in each plot. Yields were weighed after threshing, and their dry weight was determined on the basis of a grain moisture content of 14%. Maize moisture content was measured using a PM-8188-A grain moisture meter (Sanfeng precision measuring instrument Co., Ltd., Zhongshan, China). And spike length, spike diameter, kernels per ear and 100-kernels weight were measured in the laboratory.

### 5.4. Compensation Effect of Soil Accumulated Temperature on Air Accumulated Temperature

A normal process of crop growth and development requires a certain level of accumulated temperature (also termed “growing degree days (GDD)”), which, upon reaching a certain value, determines the fulfillment of a certain growth period [25]. According to the description in “Crop Cultivation Science” [32], for spring maize in the Shenyang area, the lower biological limit temperature (*T_b_*) is 8 °C and the upper biological limit temperature (*T_u_*) is 35 °C. Currently, it is common to evaluate the effective accumulated temperature as follows:

(3) $$T_{cum}=\sum_{n=1}^{n=i}\left(T-T_{b}\right)$$

where *T_cum_* is the effective accumulated temperature (°C d), and *T_i_* refers to the daily average air temperature (*T_a_*) or the daily average soil surface temperature (*T_s_*) (°C). 

When *T_i_* exceeds 35 °C, it is calculated at 35 °C, and when *T_i_* is lower than 8 °C, it is calculated at 8 °C [32].

The maize growth rate is mainly affected by the soil temperature at 5 cm depth [27], which can be used to evaluate the thermal effect of different mulching treatments. The maize growth period under film mulching is shorter than under no mulching, because the increase in soil temperature due to film mulching makes up for the low effective air temperature. Thus, the maize warming compensation coefficient (*K*) can be evaluated as follows [33]:

(4) $$K=\frac{T_{cum-a-AL}-T_{cum-a-FM}}{T_{cum-s-}{}_{FM}-T_{cum-s-}{}_{AL}}$$

where *T_cum-a-AL_* is the effective accumulated air temperature under no mulching (°C d), *T_cum-a-FM_* is the effective accumulated air temperature under film mulching (°C d), *T_cum-s-FM_* is the effective accumulated soil temperature under film mulching (°C d), and *T_cum-s-AL_* is the effective accumulated soil temperature under no mulching when the time is the same as film mulching (°C d).

Δ*T* is the compensation value of the accumulated air temperature for every 1 °C increase in the accumulated soil temperature under film mulching compared to that under no mulching. It is calculated using an empirical formula (with *K* from Equation (4)) as follows:

(5) $$\Delta T=\frac{K\times (T_{s-FM}-T_{s-AL})(T_{a}-T_{b})}{\left(T_{s-AL}-T_{b}\right)}$$

Here *T_s-FM_* is the daily average soil temperature at 5 cm under film mulching (°C), and *T_s-AL_* is the daily average soil temperature at 5 cm under no mulching (°C). Generally, a larger ratio of soil temperature to air temperature results in greater compensation. 

*T_a-FM_*, the daily average effective air temperature after the compensation of accumulated soil temperature under film mulching (°C) is related to *T_a-AL_*, the daily average effective air temperature under no mulching (°C), through:

(6) $$T_{a-FM}=T_{a}+\Delta T$$

According to the study of Sun et al. [13], the compensation effect of soil temperature on air temperature after maize tasseling is negligible. The compensatory coefficient for effective accumulated air temperature of plastic film mulching is shown in Table 10.

### 5.5. Brief Introduction of the Logistic Equation

The logistic equation (also called the “logistic curve”) was proposed by the Belgian mathematician Verhulst to describe population growth, and, in the context of crop modeling, it can be used to describe plant growth as follows:

(7) $$y=c/\left(1+e^{a+bt}\right)$$

where *y* is plant height, leaf area index or DMA, etc, *t* is the number of days after emergence, and *a, b* and *c* are fitting coefficients.

In this study, Origin 2016 software was used to fit the relationship between the DMA of spring maize and the effective accumulated air temperature compensated by effective accumulated soil surface temperature. It was found that the trend of the logistic curve was similar to the trend of the measured data. The optimized logistic equation was as follows:

(8) $$y=a/\left(1+e^{-k\left(x-x_{c}\right)}\right)$$

where *y* is the DMA, *x* is the effective accumulated temperature, and *a, k*, and *x_c_* are parameters (*a* is the final value of the DMA, *x_c_* is the sigmoid’s inflection point (midpoint), and *k* is the logistic growth rate (the steepness of the curve)).

Firstly, a function expression of Equation (8) is set in origin 2016 software. Then, input the measured value of DMA and the corresponding effective accumulated temperature in software, and the fitted curve and the corresponding values of *a, k* and *x_c_* can be obtained by running the set function. Next, the relevant characteristics can be calculated:

The differentiation of Equation (8) provides the rate of DMA (termed the growth rate, *GR*).

(9) $$GR=\frac{ake^{\left(-k(x-x_{c})\right)}}{{\left(1+e^{\left(-k(x-x_{c})\right)}\right)}^{2}}$$

Furthermore, equating the derivative of Equation (9) to 0 provides the accumulated temperature for which the growth rate is maximum, which is recorded as *x_inf_*:

(10) $$x_{inf}=x_{c}$$

The maximum growth rate, *GR_max_* (substituting Equation (10) into Equation (9)), can be determined as follows:

(11) $$GR_{\max}=\frac{1}{4}ak$$

Darroch and Baker [34] considered that, when the DMA reaches 95% of the final DMA, i.e., when *y* = 0.95*a*, growth practically stops. This happens when the effective accumulated temperature, *x_max_*, satisfies the following relationship (from Equation (8)):

(12) $$x_{max}=x_{c}+\frac{\ln19}{k}$$

The effective accumulated temperature at which the rate of increase in *GR* is maximum, *x_l_* (obtained by equating the second derivative of Equation (9) to 0), can be regarded as the beginning of the “fast growing period” of spring maize:

(13) $$x_{1}=x_{c}-\frac{\ln3}{k}$$

### 5.6. Evaluation of Relative Water Uptake (RWU) Dependence on Plant Density

The effect of PD on plant growth and yield was evaluated via also the universal Y–PD relationship proposed by Friedman [14], accounting for water availability and competition among neighboring root systems, and assuming that the relative yield, i.e., the yield normalized by the maximum yield obtained at maximum PD (*Y/Y_max_*), is equal to the relative water uptake (*RWU*), i.e., the water uptake normalized by the water uptake at maximum PD. According to the Y–PD relationship, the yield for a given PD increases approximately linearly with increasing root system radius and soil capillary length (and with decreasing planting rectangularity [14], ignored in the present analysis). Consequently, the interplant competition factor is approximately equal to 4πr_0_α^−1^, i.e., to the surface area of a sphere with a radius equal to the geometric mean of the radius of the root system (*r*_0_) and the soil capillary length (*α^−^*^1^). Thus, if we accept the hypothesis regarding the correlation between relative yield (*Y/Y_max_*) and relative water availability, i.e., that water availability plays the dominant role in determining the *Y(ρ)* relationship, it takes the following universal form:

*Y*/*Y*_max_ = RWU = 1 − exp(−4*πr*_0_*α*^−1^*ρ*) (14)

where *α^−^*^1^ and *r*_0_ are measured in units of m and ρ, the plant density, in plants·m^−2^. Its dimensionless form, incorporating the dimensionless PD (P = 4*ρ*/α^2^) and the dimensionless radius of the root system (R_0_ = *αr*_0_/2), is *Y/Y_max_* = 1 − exp(−2π*R*_0_*P*).

Firstly, the interplant competition factor (4π*r*_0_*α*^−1^) was evaluated on the basis of the dependence of the fitted final DMA, *a* (kg ha^−1^), and of the maximum growth rate, *GR_max_* (kg (ha °C d)^−1^), on the PD, (plants m^−2^). Then, the fitted value of r_0_α^−1^ was used to analyze the effect of mulching on the dependence of *a, GR_max_*, and yield on the PD (i.e., *a(ρ)*, *GR_max_(ρ)* and *Y(ρ))*.

### 5.7. Statistical Analysis

Origin 2016 (OriginLab Corporation, MA, USA) and DPS 7.05 (Hangzhou Ruifeng Information Technology Co., Ltd., Zhejiang, China) were used for data analysis, and Duncan’s new complex range method was used for the significance test. Differences at *p* < 0.05 level were considered statistically significant.

## Figures and Tables

**Table 1:** Influence of different film mulching treatments on maize developmental progress, the effective accumulated soil temperature (EAST), the effective accumulated air temperature (EAAT) and the effective accumulated soil temperature under no mulching when the time is the same as plastic film mulching (EAST-NMTSM) in 2016. Different letters are significantly different at the *p* < 0.05 probability level.

Growth Stages	Mulching Treatments	Growth Time (d)	EAST (°C d)	EAAT (°C d)	EAST-NMTSM (°C d)
Sowing—Three-leaf stage	M0	21 a	199.1 a	182.4 a	
	M1	16 b	172.1 b	129.8 c	143.8 b
	M2	18 b	180.0 b	150.7 b	162.0 a
Three-leaf—Jointing stage	M0	27 a	470.2 b	373.9 a	
	M1	26 a	475.2 b	354.1 b	445.0 b
	M2	27 a	487.9 a	365.5 ab	469.7 a
Jointing—Tasseling stage	M0	24 a	388.1 b	403.9 a	
	M1	23 a	385.2 b	375.2 b	366.8 b
	M2	24 a	396.5 a	398.6 a	384.5 a
Tasseling—Silking stage	M0	16 a	251.5 b	287.3 a	
	M1	15 a	267.7 a	272.0 b	258.2 a
	M2	15 a	254.2 b	270.7 b	246.5 b
Silking—Maturity stage	M0	52 a	783.8 a	854.4 a	
	M1	50 a	809.8 a	850.5 a	789.5 a
	M2	52 a	808.1 a	857.9 a	790.7 a
Total growth season	M0	140 a	2092.7 b	2101.8 a	
	M1	130 c	2110.1 ab	1981.5 c	2003.3 b
	M2	136 b	2126.7 a	2043.4 b	2053.3 a

**Table 2:** Influence of different film mulching treatments on maize developmental progress, the effective accumulated soil temperature (EAST), the effective accumulated air temperature (EAAT) and the effective accumulated soil temperature under no mulching when the time is the same as plastic film mulching (EAST-NMTSM) in 2017. Different letters are significantly different at the *p* < 0.05 probability level.

Growth Stages	Mulching Treatments	Growth Time (d)	EAST (°C d)	EAAT (°C d)	EAST-NMTSM (°C d)
Sowing—Three-leaf stage	M0	20 a	240.8 a	202.5 a	
	M1	14 b	183.7 c	126.2 c	163.1 b
	M2	16 b	207.7 b	155.2 b	189.1 a
Three-leaf—Jointing stage	M0	28 a	462.7 b	373.5 a	
	M1	27 a	474.8 a	336.9 b	440.2 a
	M2	27 a	470.6 ab	338.1 b	443.9 a
Jointing—Tasseling stage	M0	23 a	420.1 a	427.1 a	
	M1	22 a	421.3 a	392.4 b	397.6 a
	M2	22 a	417.9 a	403.7 b	403.2 a
Tasseling—Silking stage	M0	15 a	265.2 b	283.6 b	
	M1	14 a	277.9 ab	287.3 b	267.2 b
	M2	15 a	283.6 a	298.6 a	279.1 a
Silking—Maturity stage	M0	56 a	785.0 b	859.0 b	
	M1	55 a	834.4 a	903.9 a	815.1 a
	M2	55 a	826.1 a	880.6 ab	804.8 b
Total growth season	M0	142 a	2173.6 b	2145.5 a	
	M1	132 b	2192.1 ab	2046.6 b	2083.2 b
	M2	135 ab	2205.8 a	2076.1 b	2120.1 a

**Table 3:** Variance analysis of yield and its components under different treatments. The data in the table are F values. * indicates significantly different at *p* < 0.05 level. ** indicates significantly different at *p* < 0.01 level.

Source of Variation	Freedom Degrees	Year	Factor	Yield	Spike Length	Spike Diameter	Kernels per Ear	100-Kernels Weight
Block	2	2016	Mulching	6.134 *	5.068 *	0.221	10.695 **	7.732 **
Mulching	2		Plant density	13.047 **	7.935 **	1.592 *	8.854 **	16.126 **
Error	4		Mulching × Plant density	2.824 *	3.572 *	0.095	3.12 *	6.680 **
Plant density	4	2017	Mulching	5.572 *	4.489 *	1.096	2.726 *	9.925 **
Mulching × Plant density	8		Plant density	11.694 **	7.187 **	4.270 **	5.744 **	11.463 **
Error	24		Mulching × Plant density	1.109	3.282 *	0.849	0.571	0.932
The sum	44							

**Table 4:** Effects of different treatments on yield of spring maize and its components. Different letters are significantly different at the *p* < 0.05 probability level.

Year	Treatments	Yield (kg ha^−1^)	Spike Length (cm)	Spike Diameter (cm)	Kernels per Ear	100-Kernels Weight (g)
2016	M0	12,286.4 b	16.8 b	49.7 a	639.4 c	34.3 c
	M1	12,855.7 a	17.0 a	49.8 a	659.0 b	36.5 b
	M2	13,224.6 a	17.1 a	49.9 a	672.6 a	37.0 a
	D1	11,148.7 d	17.5 a	50.5 a	671.9 a	37.2 a
	D2	12,176.7 c	17.2 ab	49.7 ab	675.8 a	35.7 c
	D3	13,042.5 b	17.1 b	49.8 ab	661.2 b	36.0 b
	D4	13,964.0 a	16.7 c	49.7 ab	650.6 c	35.6 c
	D5	13,612.5 a	16.6 c	49.2 b	625.4 d	35.2 d
2017	M0	11,837.3 b	16.2 b	48.4 a	583.3 b	31.4 b
	M1	12,356.4 ab	16.5 ab	48.7 a	603.6 ab	32.8 ab
	M2	12,816.9 a	16.7 a	48.9 a	615.1 a	34.3 a
	D1	10,971.6 c	17.2 a	49.9 a	636.4 a	34.8 a
	D2	11,454.8 c	16.8 ab	49.3 ab	623.1 a	33.7 ab
	D3	12,556.4 b	16.8 ab	49.2 ab	616.2 a	33.5 ab
	D4	13,434.9 a	16.2 b	48.4 b	596.0 b	32.0 b
	D5	13,266.8 a	15.6 c	46.5 c	561.6 c	30.1 c

**Table 5:** Logistic curve parameters (*a*, *k*, and *x_c_*) and statistic test (*p* and *R*^2^) of the dry matter accumulation of spring maize.

Treatments	2016					2017				
	*a* (kg ha^−1^)	*k* (°C d)^−1^	*x_c_* (°C d)	*p*	*R* ^2^	*a* (kg ha^−1^)	*k* (°C d)^−1^	*x_c_* (°C d)	*p*	*R* ^2^
M0D1	26,557	0.0025	1354.9	0.006	0.97	18,978	0.0098	1185.9	0.013	0.96
M0D2	29,637	0.0022	1370.9	0.001	0.99	24,361	0.0071	1248.2	0.020	0.94
M0D3	30,453	0.0026	1223.3	0.001	0.99	28,389	0.0063	1297.9	0.019	0.94
M0D4	30,796	0.0028	1128.4	0.003	0.98	29,037	0.0054	1330.1	0.030	0.91
M0D5	34,542	0.0021	1390.7	0.006	0.97	27,744	0.0051	1328.6	0.037	0.89
M1D1	28,526	0.0020	1425.9	0.001	0.99	24,340	0.0060	1437.2	0.029	0.91
M1D2	31,079	0.0019	1495.8	0.001	0.99	28,920	0.0058	1480.9	0.021	0.94
M1D3	30,356	0.0019	1271.6	0.001	0.99	33,946	0.0064	1483.1	0.011	0.97
M1D4	32,744	0.0019	1287.8	0.002	0.98	28,767	0.0078	1411.1	0.009	0.97
M1D5	34,409	0.0018	1463.8	0.007	0.95	28,114	0.0085	1380.9	0.010	0.97
M2D1	27,485	0.0024	1314.5	0.002	0.99	26,416	0.0061	1418.2	0.018	0.95
M2D2	29,730	0.0025	1277.0	0.004	0.98	28,409	0.0057	1442.6	0.018	0.95
M2D3	35,639	0.0020	1431.1	0.001	0.99	32,887	0.0062	1439.2	0.011	0.97
M2D4	36,214	0.0020	1311.0	0.007	0.96	36,660	0.0059	1463.0	0.011	0.97
M2D5	40,185	0.0019	1442.2	0.006	0.96	35,815	0.0068	1413.6	0.008	0.98
*C_v_*	11.6%	14.5%	7.4%			16.0%	19.1%	6.4%		

**Table 6:** Variance analysis of dynamic characteristic parameters of dry matter accumulation under different treatments. The data in the table are F values. * indicates significantly different at *p* < 0.05 level. ** indicates significantly different at *p* < 0.01 level.

Source of Variation	Freedom Degrees	Year	Factor	*a*	*x_inf_*	*GR_max_*	*x_max_*	*x*_1_
Block	2	2016	Mulching	12.404 *	7.427 **	2.244 *	2.449 *	1.489
Mulching	2		Plant density	13.744 **	0.771	6.489 *	0.879	8.644 **
Error	4		Mulching × Plant density	3.693 *	0.402	4.997 *	0.407	0.988
Plant density	4	2017	Mulching	15.489 **	6.096 **	1.669	1.246	1.894
Mulching × Plant density	8		Plant density	14.221 **	1.204	5.744 *	1.042	6.884 *
Error	24		Mulching × Plant density	2.109 *	0.587	4.849 *	0.946	1.032
The sum	44							

**Table 7:** Effects of different treatments on dynamic characteristic parameters of dry matter accumulation. Different letters are significantly different at the *p* < 0.05 probability level.

Year	Treatments	*a* (kg ha^−1^)	*x_inf_* (°C d)	*GR_max_* (kg (ha °C d) ^−1^)	*x_max_* (°C d)	*x*_1_ (°C d)
	M0	30,397.2 c	1293.6 b	18.5 a	2513.4 b	838.5 a
	M1	31,422.6 b	1389.0 a	14.8 b	2953.3 a	805.3 a
	M2	33,850.5 a	1355.2 a	18.0 a	2734.2 a	840.7 a
2016	D1	27,522.5 e	1365.09 a	15.7 c	2667.5 a	874.4 a
	D2	30,148.8 d	1381.3 a	16.5 bc	2740.1 a	879.1 a
	D3	32,149.3 c	1308.7 a	17.2 ab	2704.5 a	787.9 b
	D4	33,251.3 b	1242.4 a	18.3 a	2606.8 a	733.3 c
	D5	36,378.6 a	1432.2 a	17.7 a	2949.4 a	866.2 a
	M0	25,701.8 c	1278.1 b	41.8 a	1739.0 a	1106.2 a
	M1	28,817.6 b	1438.6 a	49.8 a	1874.4 a	1276.0 a
	M2	32,037.5 a	1435.3 a	49.3 a	1916.6 a	1255.7 a
2017	D1	23,244.5 d	1347.1 a	41.2 c	1770.5 a	1232.7 a
	D2	27,230.1 c	1390.5 a	41.9 c	1869.8 a	1224.2 ab
	D3	31,740.9 a	1406.7 a	49.9 b	1874.9 a	1211.0 b
	D4	31,488.1 a	1401.4 a	49.7 b	1876.4 a	1189.1 d
	D5	30,558.0 b	1374.4 a	52.1 a	1825.2 a	1206.2 c

**Table 8:** Path analysis of the final dry matter accumulation (*a*) and the characteristics of the dynamic process. ** indicates significant correlation at *p* < 0.01 level.

Characteristic Parameters	Correlation Coefficient	Direct Path Coefficient	Indirect Path Coefficient				The Contribution Rate to *R*^2^
			*x_inf_*	*GR_max_*	*x_max_*	*x*_1_	
*x_inf_*	0.32	0.1967		0.2643	−0.1066	−0.0301	0.0629
*GR_max_*	0.96 **	1.0072	0.0116		−0.07	0.0049	0.9669
*x_max_*	0.53	−0.1133	0.215	0.50		−0.0717	−0.0600
*x*_1_	0.03	−0.0979	0.2609	−0.0503	−0.083		−0.0029

**Table 9:** The physical properties of soil in the experimental site.

Soil Layer	Particle Composition (%)			Soil Texture
(cm)	<0.002 mm	0.002–0.05 mm	0.05–2 mm	(International System)
0–40	11.6	60.5	27.9	silty loam
40–100	18.9	58.0	23.1	silty clay loam

**Table 10:** Compensatory coefficient for accumulated air temperature of plastic film mulching.

Year	Treatments	Sowing—Three-Leaf Stage	Three-Leaf—Jointing Stage	Jointing—Tasseling Stage
2016	M1	1.86	0.65	1.56
	M2	1.76	0.46	0.44
2017	M1	3.71	1.06	1.46
	M2	2.54	1.32	1.59

## Data Availability

The data presented in this study are available on request from the corresponding author.
